# Key mechanisms of plant-*Ralstonia solanacearum* interaction in bacterial wilt pathogenesis

**DOI:** 10.3389/fmicb.2025.1521422

**Published:** 2025-06-06

**Authors:** Zaid Chachar, Xiaoming Xue, Junteng Fang, Ming Chen, Chen Jiarui, Weiwei Chen, Nazir Ahmed, Sadaruddin Chachar, Mehar-un-Nisa Narejo, Naseer Ahmed, Lina Fan, Ruiqiang Lai, Yongwen Qi

**Affiliations:** ^1^College of Agriculture and Biology, Zhongkai University of Agriculture and Engineering, Guangzhou, China; ^2^Institute of Nanfan and Seed Industry, Guangdong Academy of Science, Guangzhou, China; ^3^College of Horticulture and Landscape Architecture, Zhongkai University of Agriculture and Engineering, Guangzhou, China; ^4^Department of Crop Physiology, Sindh Agriculture University, Tandojam, Pakistan; ^5^Institute of Grassland Research (IGR), Chinese Academy of Agricultural Sciences, Hohhot, China

**Keywords:** *Ralstonia solanacearum*, bacterial wilt, virulence factors, plant immune response, disease resistance

## Abstract

*Ralstonia solanacearum*, a Gram-negative bacterium, is the causative agent of bacterial wilt, a devastating disease affecting a wide range of economically important crops worldwide. This study explores the dynamic interactions between *Ralstonia solanacearum* and its host plants, emphasizing key mechanisms underlying infection and host response. The pathogen initiates infection through root wounds or natural openings, rapidly colonizing xylem vessels where it forms biofilms that disrupt water and nutrient transport. Its virulence is driven by cell wall-degrading enzymes and effector proteins delivered via a Type III secretion system, which subvert plant immune responses and facilitate systemic spread. In turn, host plants activate hormonal and stress-related defense pathways, though these are often manipulated by the pathogen, leading to disease progression and reduced productivity. This review highlights critical gaps in our understanding of molecular host-pathogen interactions and the role of environmental conditions in disease development. Addressing these gaps is vital for improving management strategies, with breeding for resistance and advanced biotechnological tools offering promising solutions to combat bacterial wilt and support sustainable agriculture. Future research should focus on leveraging genetic insights to enhance host resistance, employing advanced biotechnological tools to develop crop varieties with enhanced resistance to *Ralstonia solanacearum*, thereby promoting sustainable agriculture and strengthening global food security.

## 1 Introduction

*Ralstonia solanacearum* is a highly aggressive soil-borne bacterium that causes bacterial wilt in major crops including tomato, potato, eggplant, and banana. With the capacity to infect over 310 species across 42 plant families, it ranks among the most widespread and adaptable plant pathogens (Wang et al., 2023b). Its pathogenicity is primarily driven by colonization of the vascular system and secretion of Type III effectors that suppress host immunity and enable systemic infection (Moussa et al., 2022).

The extensive host range and genetic variability of *R. solanacearum* make it particularly difficult to manage. These characteristics contribute to its adaptability and persistence across diverse agricultural systems. Recent studies have identified key virulence factors, including effector proteins and biofilm formation, that contribute to its pathogenicity and persistence in the environment (Vargas et al., 2023). Understanding these mechanisms is crucial for developing resistant cultivars and integrated disease management strategies (Jang and Seo, 2023).

This review examines how *Ralstonia solanacearum* infects plants and avoids their immune defenses, highlighting the key biological processes that enable its success as a pathogen. A major aim is to examine the molecular details of the pathogen’s virulence factors, including effector proteins and cell wall-degrading enzymes, which facilitate successful infection. Understanding these processes can help identify novel targets for control strategies, moving beyond current treatment options that are often ineffective due to the pathogen’s persistence and genetic diversity. Further explores the significance of biofilm formation in pathogenesis, which complicates treatment efforts and contributes to the pathogen’s resilience. Additionally, the interaction between *Ralstonia solanacearum* and host plant stress responses will be analyzed, with an emphasis on the hormonal and physiological adaptations that occur during infection. This analysis seeks to address the knowledge gaps in the molecular mechanisms underlying pathogen-host interactions and the environmental factors influencing disease dynamics.

This review evaluates the effectiveness of current control strategies for *Ralstonia solanacearum*, focusing on breeding resistance, leveraging genetic insights, and developing biotechnological tools to enhance host resistance. These approaches aim to improve agricultural productivity and food security in the face of this challenging pathogen. We also examine the multifaceted interactions between *Ralstonia solanacearum* and its host plants, highlighting the pathogen’s virulence factors, including effector proteins and enzymes that degrade plant cell walls, facilitating infection. Understanding how *Ralstonia solanacearum* evades plant immune responses and forms biofilms will provide insights into novel control strategies. Furthermore, analyzing plant stress signaling pathways and physiological adaptations during infection can identify potential targets for future interventions.

This review aims to provide a comprehensive examination of the complex interactions between *Ralstonia solanacearum* and its host plants, focusing on the bacterium’s entry, colonization, and systemic spread. It delves into the mechanisms by which the pathogen employs virulence factors such as cell wall-degrading enzymes and effector proteins, mediated through a Type III secretion system, to manipulate host cellular processes and suppress immune responses. The review also discusses the activation of stress-related pathways and hormonal changes within host plants, showing how the bacterium’s manipulation leads to compromised plant health and reduced productivity. A critical aspect of this review is identifying gaps in current knowledge related to the molecular details of pathogen-host interactions and the influence of environmental factors on disease dynamics. By highlighting these gaps, the review underscores the potential for developing effective management strategies, emphasizing breeding for resistance as a promising approach. Future research directions include leveraging genetic insights to enhance host resistance and employing advanced biotechnological tools to develop crop varieties resilient against *Ralstonia solanacearum* infections, ultimately aiming for sustainable agricultural practices and improved food security.

## 2 Challenges and control of *Ralstonia solanacearum* in plants

Managing *Ralstonia solanacearum* poses significant challenges. Its wide host range, ability to persist in diverse environments, and rapid systemic spread through plant vasculature complicate effective control (Chen Y. et al., 2023). Effective control requires an integrated approach combining cultural, biological, and genetic strategies, particularly in resource-constrained regions (Wang et al., 2023b).

Cultural practices, including crop rotation with non-host species and the application of soil amendments, play a critical role in suppressing *R. solanacearum* populations. For instance, rotation with cereals and the use of organic matter have been shown to enhance soil microbial diversity and reduce disease incidence in smallholder farming systems (Jones, 2021). Biological control using antagonistic microbes e.g., *Bacillus subtilis* and *Trichoderma* spp. can reduce disease severity by producing antimicrobial metabolites and inducing systemic resistance (Jaiswal et al., 2022).

Integrated Pest Management (IPM), a holistic approach that integrates cultural, biological, and genetic strategies, combines resistant cultivars, biocontrol agents, and improved agronomic practices. These systems have demonstrated region-specific success in reducing disease pressure and enhancing crop productivity (Galli et al., 2024). For example, in Latin America, IPM adoption with crop rotation, biofertilizers, and marker-assisted-selected varieties led to reduced bacterial wilt incidence and input costs. However, effectiveness varies with environmental conditions and pathogen diversity, necessitating localized solutions and continuous monitoring. In Latin America, farmers adopted integrated practices such as rotating tomato crops with non-host plants, applying biofertilizers containing *Pseudomonas fluorescens*, and cultivating resistant varieties developed through marker-assisted selection to manage bacterial wilt more effectively. This integrated approach not only reduced disease incidence but also minimized chemical input costs, supporting ecological and economic sustainability (Galli et al., 2024).

Crop rotation with non-host plants, such as cereals, can effectively disrupt the disease cycle and reduce pathogen buildup in the soil (Jones, 2021). Soil health management, including the use of organic amendments and conservation tillage, has been shown to suppress *R. solanacearum* populations and enhancing beneficial microbial communities. For instance, long-term studies in smallholder farming systems in Southeast Asia demonstrated that integrating cover crops like legumes with rotation schemes significantly reduced bacterial wilt incidence (Kumar, 2018). Biological Strategies: Beneficial microorganisms, including bacterial and fungal biocontrol agents, play a crucial role in integrated pest management (IPM). For example, *Bacillus subtilis* and *Trichoderma* spp. have shown efficacy in suppressing *R. solanacearum* by producing antimicrobial compounds and enhancing plant systemic resistance. Case studies in East Africa revealed that the application of these biocontrol agents, combined with composted organic matter, improved crop yields and reduced disease severity by over 50%. Additionally, incorporating practices like the use of green manures and essential oils derived from plants such as neem and marigold has proven effective in smallholder farming contexts (Jaiswal et al., 2022).

By expanding the focus to include practical case studies and successful applications of cultural and biological strategies, this review underscores their importance, especially for smallholder farmers in resource-limited settings. Future research should prioritize scalable IPM solutions that combine these strategies with emerging biotechnological tools to address the diverse needs of global agricultural systems.

### 2.1 Biological approaches

Biological control strategies utilize natural antagonists microorganisms, plant-based compounds, and organic amendments to inhibit the growth and spread of *Ralstonia solanacearum*. Among the most promising are biocontrol agents (BCAs), including bacteria, fungi, and bacteriophages (Ayaz et al., 2023). Bacterial BCAs such as *Bacillus* spp., *Pseudomonas* spp., and *Paenibacillus* spp. act through multiple mechanisms: producing antimicrobial metabolites, competing for resources, and priming host plant defenses. For example, *Bacillus velezensis* improves tomato resistance by releasing lipopeptides and volatile organic compounds. Similarly, fungal agents like *Trichoderma* spp. secrete enzymes that degrade the bacterial cell wall, reducing pathogen viability. Bacteriophages, though still in the early stages of experimental application, offer targeted control by directly lysing *R. solanacearum* cells (Ye et al., 2022).

Organic amendments such as composts, green manures, and plant residues enhance soil microbial diversity and indirectly suppress *R. solanacearum* by creating unfavorable conditions for its proliferation. Essential oils and plant extracts have shown antibacterial activity, although their field efficacy depends on soil type, climate, and amendment composition.

**FIGURE 1:**
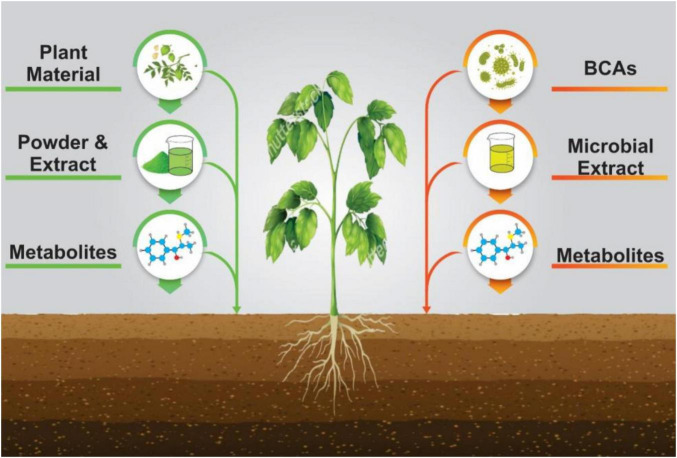
Biological strategies for controlling *Ralstonia solanacearum*, incorporating plant-based approaches and biological control agents (BCAs). These methods form a crucial part of Integrated Disease Management (IDM).

Together, microbial BCAs and plant-derived products form a key component of Integrated Disease Management (IDM). Their eco-friendly nature makes them especially valuable for smallholders and organic farming systems seeking to reduce dependence on synthetic pesticides (Figure 1). The main objective of these approaches is to minimize or eliminate the use of synthetic pesticides, offering an eco-friendly solution for pest management. Selection of these approaches depends on factors like the host plant, environmental conditions, the specific pathogen, and its life cycle. For managing *Ralstonia solanacearum*, two major biological strategies are emphasized: biocontrol agents (BCAs) and plant-based organic products. BCAs include live microbial agents, microbial extracts, and their metabolites. These work through mechanisms such as competitive exclusion, antibiosis, and the activation of plant defenses. On the other hand, plant-based products, such as raw plant materials, powders, extracts, and plant metabolites, contribute to plant defense mechanisms by enhancing immunity and directly inhibiting pathogen growth. Together, these biological strategies offer an integrated approach to managing *R. solanacearum*, contributing to environmentally sustainable farming by reducing dependence on chemical pesticides.

### 2.2 Breeding and genetic engineering approaches

Developing crop resistance to *Ralstonia solanacearum* is essential for sustainable disease management, especially in key crops like tomato, potato, and eggplant. However, efforts are hindered by the pathogen’s genetic diversity, the polygenic nature of resistance traits, and the evolution of virulent strains capable of overcoming known resistance loci. In many crops, resistance is controlled by quantitative trait loci (QTLs), which require extensive evaluation and multiple breeding cycles, making conventional breeding a lengthy and challenging process. For instance, combining broad-spectrum and strain-specific QTLs has shown promise in potato, but requires careful selection and long breeding cycles (Chitwood-Brown et al., 2021). Furthermore, pathogen evolution can erode resistance durability, necessitating continuous screening for new resistance sources. The presence of multiple races and biovars of *R. solanacearum* complicates the development of universally resistant varieties. Additionally, resistance is often controlled by quantitative trait loci (QTLs), which may require extensive breeding efforts and marker-assisted selection. Furthermore, resistance conferred by certain genes can be overcome by pathogen evolution, necessitating continuous research to identify durable resistance sources and integrate them into breeding programs (Vargas et al., 2023). While traditional breeding methods have been successful in developing resistant varieties, the process can be slow due to the intricate inheritance of resistance traits and the necessity for multiple generations to achieve desired resistance levels (Moussa et al., 2022). Studies have identified major and minor resistance quantitative trait loci (QTLs) in potatoes, highlighting the potential for combining broad-spectrum and strain-specific QTLs to develop highly effective resistant cultivars (Chitwood-Brown et al., 2021). Additionally, research has shown the importance of soil microbiome in conferring resistance, with resistant cultivars recruiting beneficial rhizosphere bacteria that can offer protection against These findings highlight the importance of ongoing breeding efforts. They also emphasize the need to explore innovative strategies that can strengthen resistance programs and support long-term disease management.

Biotechnological tools, especially CRISPR/Cas9, now enable targeted genome editing to enhance host resistance. This includes knocking out susceptibility genes or inserting resistance genes from other species. For example, overexpression of the NPR1 gene from Arabidopsis in tomato boosts systemic acquired resistance, reducing disease incidence. Similarly, transgenic rice expressing Xa21 shows enhanced resistance not only to Xanthomonas but also to R. solanacearum, illustrating cross-species potential (Lin et al., 2004). Stacking multiple resistance genes and using marker-assisted selection (MAS) can speed up the development of robust, disease-resistant cultivars (Parihar and Shiwani, 2022)., as highlighted in various research papers (Rato et al., 2021). For example, the introduction of the Arabidopsis NPR1 gene into tomatoes has demonstrated a significant reduction in bacterial wilt incidence by enhancing systemic acquired resistance, showcasing the potential of genetic engineering in enhancing plant immunity (Lin et al., 2004). Moreover, overexpression of resistance genes like Xa21 in rice has shown promise in conferring broad-spectrum resistance against bacterial pathogens, indicating the versatility and efficacy of CRISPR/Cas9 in developing disease-resistant crop varieties with improved agricultural sustainability and productivity (Lin et al., 2004). Combining traditional breeding with modern biotechnological tools can potentially overcome the limitations of each approach. Marker-assisted selection (MAS) can expedite the breeding process by identifying and selecting for resistance traits at the molecular level. Moreover, stacking multiple resistance genes through genetic engineering can provide robust and durable resistance against *R. solanacearum* (Parihar and Shiwani, 2022).

Integrating classical breeding, genetic engineering, and microbial biocontrol strategies can reduce pesticide dependency and improve crop resilience. However, successful application requires an in-depth understanding of host-pathogen interactions and effective delivery systems for engineered traits or biocontrol agents. Tailoring these tools to local farming conditions will be essential for global adoption. However, the successful implementation of these strategies requires a thorough understanding of plant-pathogen interactions, effective delivery systems for biocontrol agents, and the development of resistant crop varieties that meet agronomic and consumer preferences.

Table 1 presents a diverse range of plants evaluated for their antibacterial activity against *Ralstonia solanacearum*, the pathogen responsible for bacterial wilt. It includes over 40 plant species from various families such as Myrtaceae, Fabaceae, Apocynaceae, and Poaceae. The studies cited explore different plant parts—mainly leaves, shoots, and rhizomes—tested either *in vitro* or on specific hosts like tomato, potato, tobacco, and hot pepper. This compilation highlights the potential of botanicals and some microbial agents as alternatives for managing bacterial wilt across different crops.

**TABLE 1:** Plants tested for their antibacterial potential against *Ralstonia solanacearum* and management of bacterial wilt disease in different hosts.

Scientific name	Common name	Family	Plant part used	Host	References
*Psidium guajava*	Lemon guava	Myrtaceae	Leaves	*In vitro*	Sandeep and Vegetos, 2009
*Piper guineense*	Benin pepper	Piperaceae	Leaves	*In vitro*	Sandeep and Vegetos, 2009
*Tagetes patula*	French marigold	Asteraceae	Leaves	*In vitro*	Din et al., 2016
*Calotropis procera*	Giant milkweed	Apocynaceae	Leaves	*In vitro*	Din et al., 2016
*Adhatoda vasica*	Malabar nut	Acanthaceae	–	*In vitro*	Din et al., 2016
*Xanthium strumarium*	Rough cocklebur	Asteraceae	Shoot	Tomato	Khan et al., 2020
*Allium sativum*	Garlic	Amaryllidaceae	–	Tomato	Abo-Elyousr and Asran, 2009
*Datura*	Datura	Solanaceae	Leaves	*In vitro*	Abo-Elyousr and Asran, 2009
*Nerium oleander*	Oleander	Apocynaceae	Shoots	*In vitro*	Abo-Elyousr and Asran, 2009
*Amanita phalloides*	Mushroom	Amanitaceae	Shoots	Potato	Erjavec et al., 2016
*Clitocybe geotropa*	Trooping funnel	Tricholomataceae	Seeds	Potato	Erjavec et al., 2016
*Hibiscus sabdariffa*	Roselle	Malvaceae	–	Potato	Hassan et al., 2009
*Punica granatum*	Pomegranate	Lythraceae	Leaves	Potato	Hassan et al., 2009
*Eucalyptus globulus*	Blue gum	Myrtaceae	–	Potato	Hassan et al., 2009
*Syringa oblata*	Broadleaf lilac	Oleaceae	Stem	Tobacco	Bai et al., 2016
*Punica granatum*	Pomegranate	Lythraceae	–	Potato	Farag et al., 2015
*Acacia*	Wattles	Fabaceae	–	Potato	Farag et al., 2015
*Eichhornia crassipes*	Water hyacinth	Pontederiaceae	Leaves	Tomato	Alemu et al., 2013
*Mimosa diplotricha*	Nila grass	Fabaceae	–	*In vitro*	Alemu et al., 2013
*Lantana camara*	Lantana	Verbenaceae	–	*In vitro*	Alemu et al., 2013
*Prosopis juliflora*	Mesquite	Fabaceae	Leaves	*In vitro*	Alemu et al., 2013
*Burcea antidysenterica*	Burcea	Burcea	Leaves	Hot pepper	Yihune and Yemata, 2019
*Eucalyptus citriodora*	Lemon gum	Myrtaceae	Leaves	Hot pepper	Yihune and Yemata, 2019
*Justicia schimperiana*	Agewgna	Acanthaceae	Leaves	Hot pepper	Yihune and Yemata, 2019
*Lantana camara*	Lantana	Verbenaceae	Leaves	Hot pepper	Yihune and Yemata, 2019
*Melia azedarach*	Chinaberry	Meliaceae	Leaves	Hot pepper	Yihune and Yemata, 2019
*Ricinus communis*	Castor bean	Euphorbiaceae	Leaves	Hot pepper	Yihune and Yemata, 2019
*Curcuma amada*	Mango ginger	Zingiberaceae	Rhizome	*In vitro*	Karthika et al., 2017
*Ocimum gratissimum*	Clove basil	Lamiaceae	–	Tomato	Kiran Kumar et al., 2017
*Tylophora asthmatica*	Antamool	Apocynaceae	Leaves	Tomato	Kiran Kumar et al., 2017
*Calotropis gigantea*	Crown flower	Apocynaceae	–	Tomato	Kiran Kumar et al., 2017
*Ocimum sanctum*	Holy basil	Lamiaceae	Leaves	Tomato	Kiran Kumar et al., 2017
*Tylophora asthmatica*	Indian ipecacuan	Asclepiadaceae	Leaves	Tomato	Kiran Kumar et al., 2017
*Nigella sativa*	Black cumin	Ranunculaceae	Shoot	Tomato	Kiran Kumar et al., 2017
*Ruta graveolens*	Rue	Rutaceae	–	Tomato	Kiran Kumar et al., 2017
*Pernettya prostrata*	Yuruñiwi	Ericaceae	–	*In vitro*	Ordóñez et al., 2023
*Rubus roseus*	Roseus Raspberry	Rosaceae	–	*In vitro*	Ordóñez et al., 2023
*Streptomyces* sp. *NEAU-HV9*	–	Streptomycetaceae	–	Tomato	Ling et al., 2020
*Clematis lasiandra*	Lasiandra	Ranunculaceae	–	*In vitro*	Tian et al., 2013
*7-Methoxycoumarin*	Various plants	Various	–	Tobacco	Li et al., 2024
*Harmine*	Various plants	Various	–	Tobacco, tomato	Xia et al., 2023
Maize (*Zea mays*)	Maize	Poaceae	Extract	Various	Thoenen et al., 2023
Rice (*Oryza sativa*)	Rice	Poaceae	Various	Various	Patel et al., 2022
Wheat (*Triticum aestivum*)	Wheat	Poaceae	Various	Various	Dutilloy et al., 2022
Sugarcane (*Saccharum* spp.)	Sugarcane	Poaceae	Various	Various	Zang et al., 2022

## 3 Entry and colonization

*Ralstonia solanacearum* initiates infection by entering through root tips, lateral root junctions, or wounds caused by cultivation or soil organisms such as nematodes (Bhuyan et al., 2024). Once inside, the bacterium uses its flagella to migrate toward xylem vessels—the primary site of systemic colonization (Shi et al., 2023). These initial interactions reflect evolutionary adaptation, allowing the pathogen to bypass surface defenses and target internal transport systems. This ability to recognize and invade through specific entry points showcases the bacterium’s evolutionary adaptation to host plants, highlighting its adeptness in early interactions for successful colonization (Mekonnen et al., 2022). Understanding these initial interactions at a microscopic level provides insights for potential intervention strategies to disrupt the early stages of infection, potentially impeding the pathogen from reaching the vascular system and causing extensive damage (Álvarez et al., 2022).

**FIGURE 2:**
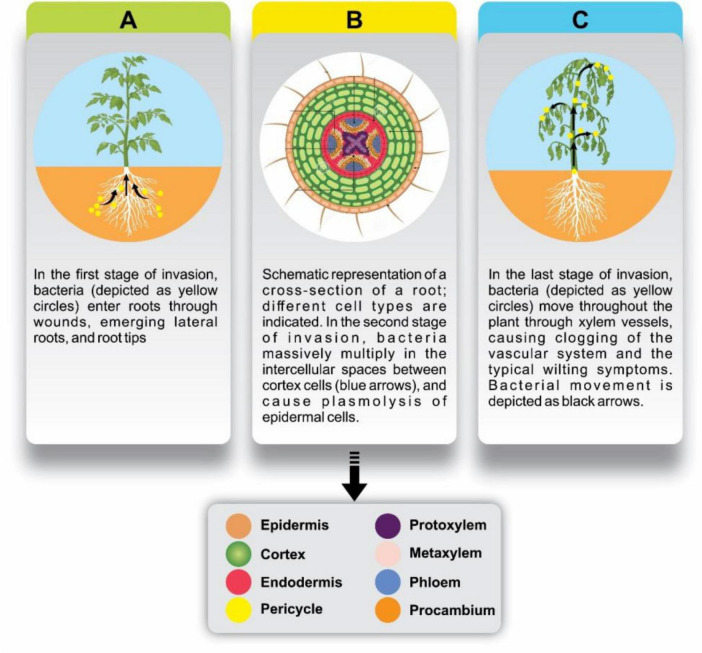
Stages of bacterial invasion in plant roots and subsequent spread through the vascular system. **(A)** In the initial invasion stage, bacteria (yellow circles) enter the roots through wounds, emerging lateral roots, and root tips. **(B)** Schematic cross-section of a root shows various cell types (legend below), highlighting the bacterial multiplication (yellow circles) in the intercellular spaces of cortex cells (blue arrows), leading to plasmolysis in epidermal cells. **(C)** In the final stage, bacteria move through xylem vessels (black arrows), causing vascular clogging and wilting symptoms in the plant.

Upon reaching the xylem (Figures 2A–C), *R. solanacearum* rapidly multiplies and forms biofilms that obstruct water and nutrient flow. The pathogen adheres to xylem walls, secretes extracellular polysaccharides (EPS), and produces enzymes that degrade host tissues, enhancing its systemic spread. These biofilms not only protect the bacteria from host immune responses but also facilitate their upward movement through the vascular system, contributing to characteristic wilt symptoms. Upon reaching the xylem (Figure 2B), the bacterium adheres to and proliferates within the plant’s vascular tissues. It secretes polysaccharides that contribute to biofilm formation, which enhances its stability and protection against plant immune responses. The subsequent systemic spread of the pathogen through xylem vessels (Figure 2C) results in vascular blockage and the characteristic wilting symptoms of infected plants. These biofilms not only secure the bacterium’s presence but also facilitate its spread upwards through the xylem, obstructing the flow of water and nutrients essential for the plant’s survival. The strategic production of extracellular enzymes during this phase further aids the bacterium in breaking down the plant’s cellular defenses and advancing its colonization efforts (Wang et al., 2023a).

This ability to rapidly colonize and establish within the xylem is what makes *Ralstonia solanacearum* particularly formidable, as it sets the stage for widespread infection and the characteristic wilting symptoms of the disease. Research into disrupting this colonization process may lead to effective strategies that limit bacterial spread within the plant and reduce disease severity.

Figure 2 illustrates the sequential stages of *Ralstonia solanacearum* invasion in plant roots, from initial entry to systemic spread. In the first stage (Figure 2A), bacteria (depicted as yellow circles) enter the plant through natural openings such as wounds, root tips, and lateral root junctions. This entry point allows the pathogen to establish itself within the root tissue. In the second stage (Figure 2B), the bacteria multiply extensively within the intercellular spaces of the cortex. A cross-sectional view of the root highlights various cell layers epidermis, cortex, and vascular tissue showing the progression of bacterial spread (indicated by blue arrows) and associated plasmolysis in epidermal cells, which compromises root integrity. In the final stage (Figure 2C), the pathogen spreads systemically via the xylem vessels (black arrows), leading to the blockage of vascular channels. This disruption in water and nutrient transport results in the characteristic wilting symptoms and physiological stress that mark advanced bacterial wilt.

The accompanying legend outlines the anatomical structure of the root, consistently labeling key tissue layers and vascular components, including the epidermis, cortex, endodermis, pericycle, and vascular tissues such as phloem (*protophloem, metaphloem*), (*cambium (procambium*), and xylem (*protoxylem, metaxylem*).

## 4 Biofilm formation and its role

Biofilm formation within the xylem vessels is central to *Ralstonia solanacearum’s* pathogenic strategy and plays a key role in the severity of bacterial wilt. These biofilms are complex, multicellular communities encased in a matrix of extracellular polymeric substances (EPS), composed of bacterial polysaccharides, proteins, and DNA (Chen Y. et al., 2023). This structure enables the bacteria to firmly adhere to plant tissues and form a protective barrier against host immune responses and environmental stresses. Within the xylem, biofilms also act as physical obstructions that disrupt the plant’s vascular flow, impeding the transport of water and nutrients. This disruption contributes significantly to the characteristic wilting symptoms. The durability and resistance of biofilms to both chemical treatments and natural plant defenses make *R. solanacearum* particularly difficult to control once established in the plant (Carezzano et al., 2023). A deeper understanding of the molecular mechanisms and genetic regulation behind biofilm formation in *Ralstonia solanacearum* could open new avenues for disease control. These biofilms are not only essential for bacterial survival and spread but also serve as a major barrier to effective treatment. By identifying key genes and pathways involved in biofilm development, researchers can design targeted interventions such as biofilm-disrupting agents that reduce the pathogen’s virulence and limit its impact on crops.

The formation of biofilms by *Ralstonia solanacearum* within xylem vessels has a profound impact on the physiology and health of the host plant. These biofilms obstruct the xylem, the plant’s primary water transport system, severely disrupting water and nutrient flow essential for survival and growth. Consequently, the plant rapidly wilts and, if left untreated, succumbs to the infection. Additionally, the biofilm serves as a reservoir for the bacteria, enabling *R. solanacearum* to persist and proliferate within the host. This persistence not only complicates disease management but also increases the risk of the pathogen spreading to other parts of the plant and to neighboring plants (Tran et al., 2016; Carezzano et al., 2023). The protective environment created by the biofilm shields the bacterial cells from environmental stresses and plant defense mechanisms, including chemical treatments like fungicides and bactericides, complicating efforts to eradicate the pathogen once biofilms have formed. The strategic advantage provided by biofilm formation highlights the necessity of targeting this aspect of the pathogen’s lifecycle in disease management strategies. Disrupting biofilm development could weaken the pathogen’s virulence and ability to spread, presenting a promising direction for future research in plant pathology (Roy et al., 2018).

**FIGURE 3:**
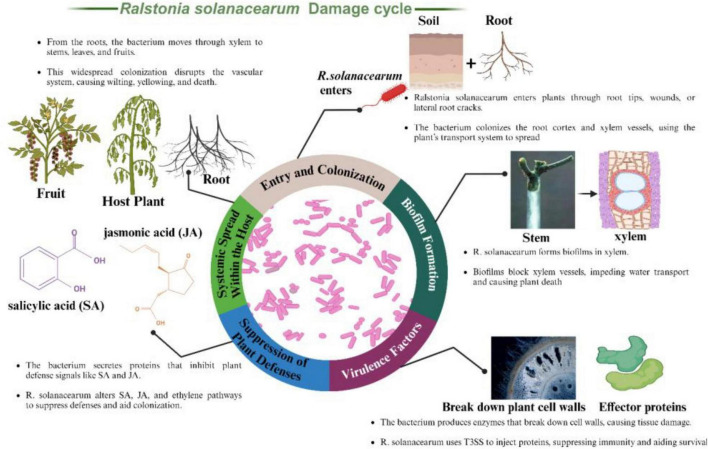
*Ralstonia solanacearum*, illustrating its entry through root tips and cracks, biofilm formation in xylem vessels, secretion of cell wall-degrading enzymes and effector proteins, suppression of plant defense pathways involving salicylic acid (SA) and jasmonic acid (JA), and systemic spread through the plant’s vascular system, leading to widespread wilting and plant death.

Figure 3 presents the infection cycle of *Ralstonia solanacearum*, highlighting how the bacterium invades and damages plant tissues. It typically enters through natural openings like root tips, wounds, or cracks near lateral roots. After entry, the pathogen colonizes the root cortex and xylem vessels key channels for water and nutrient flow. These vessels then serve as highways for the bacterium’s systemic spread, contributing to widespread wilting and plant decline. Within the xylem, *R. solanacearum* forms biofilms dense bacterial communities encased in an extracellular polymeric substance (EPS) matrix. These biofilms play a crucial role in bacterial adherence and protection against plant defenses. By blocking xylem vessels, biofilms disrupt water transport, resulting in wilting, stunted growth, and eventually plant death due to the compromised vascular system.

The bacterium employs several virulence factors to facilitate infection and spread. It produces cell wall-degrading enzymes (CWDEs) such as cellulases, pectinases, and proteases, which break down plant cell walls, aiding bacterial spread and causing tissue maceration and necrosis. Additionally, *R. solanacearum* uses a Type III secretion system (T3SS) to inject effector proteins directly into plant cells. These effectors manipulate host cellular processes, suppress immune responses, and promote bacterial survival and proliferation. To evade plant defenses, *R. solanacearum* secretes proteins that inhibit key plant defense signaling pathways involving salicylic acid (SA) and jasmonic acid (JA). By disrupting these pathways, the bacterium prevents the plant from mounting an effective immune response. Furthermore, *R. solanacearum* manipulates hormonal pathways regulated by SA, JA, and ethylene, aiding in suppressing host defenses and facilitating its colonization. After establishing an infection in the roots, *R. solanacearum* spreads systemically through the xylem vessels to other parts of the plant, including stems, leaves, and fruits. This systemic movement utilizes the plant’s vascular system, leading to widespread colonization. The extensive spread of the bacterium disrupts the plant’s vascular system, resulting in severe wilting, yellowing, and eventual death of the plant.

This comprehensive depiction of *Ralstonia solanacearum*’s infection process underscores the pathogen’s sophisticated strategies to invade, colonize, and damage its host plants, causing significant agricultural losses.

## 5 Virulence factors

The pathogenicity and systemic spread of *Ralstonia solanacearum* are driven by a broad range of virulence factors, particularly enzymes that break down plant cell walls and effector proteins that interfere with host immune responses (Chen Y. et al., 2023). While enzymes such as cellulases, pectinases, and hemicellulases are well-characterized for their roles in tissue degradation and nutrient release, the variability and specific roles of these enzymes across different *R. solanacearum* strains remain underexplored. Addressing these gaps could provide new insights into strain-specific pathogenic strategies and their implications for host adaptation (Truchon et al., 2023; Wang et al., 2023a).

The T3SS functions as a molecular syringe, delivering over 70 known effector proteins (T3Es) into host cells. These effectors manipulate host immune pathways, interfere with signaling, and enable bacterial proliferation. Their diversity explains why resistance based on single genes is often ineffective across strains (Wu et al., 2024).

### 5.1 Key effectors and their targets

• *RipG7*: Interacts with plant E3 ubiquitin ligases to degrade immune-related proteins, weakening the plant’s defense response (Langin et al., 2023).
• *RipAA (PopP2)*: Acts as an acetyltransferase that targets WRKY transcription factors involved in basal defense, leading to suppression of salicylic acid-mediated responses (Qi et al., 2022).
• *RipAY*: Cleaves glutathione, a key molecule in plant stress responses, thereby reducing oxidative burst and promoting bacterial survival (Sang et al., 2018).
• *RipTPS*: Mimics plant trehalose-6-phosphate synthase, disrupting sugar metabolism and energy allocation, making the plant more susceptible to infection (An et al., 2022).
• *RipV2*: Binds to the host BSK1 kinase, a key component of immune signaling, effectively blocking downstream defense pathways (Schreiber et al., 2021).

The diversity of *R. solanacearum* effectors highlight the complexity of host-pathogen interactions and explains why single resistance genes often fail against different bacterial strains. A deeper understanding of these effector-target interactions is crucial for designing broad-spectrum resistance strategies, including engineered resistance proteins that specifically recognize and neutralize T3SS effectors (Wang et al., 2023a). Understanding these effectors and their interactions with plant cells is a major research focus. Identifying these effectors and elucidating their mechanisms of action are critical for comprehending *R. solanacearum*’s pathogenic strategies and provide valuable targets for genetic engineering and breeding programs aimed at enhancing plant resistance to bacterial wilt. For instance, plants could be genetically modified to block these effectors’ entry or deactivate their functions, preserving the plant’s immune response integrity.

In summary, targeting key virulence factors such as cell wall-degrading enzymes and Type III secretion system effectors through breeding and genetic engineering presents a promising path toward developing resistant plant varieties. For example, engineering plants to recognize or block effector entry could preserve immune integrity. Ongoing research into effector-target interactions is critical for designing durable, multi-strain resistance.

## 6 Suppression of plant defenses

*Ralstonia solanacearum* suppresses host defenses through effector-mediated disruption of immune signaling. By interfering with recognition systems and hormonal defense pathways, the pathogen evades early detection and establishes systemic infection (Shi et al., 2023). Its ability to evade detection often stems from effectors that mask pathogen-associated molecular patterns (PAMPs) conserved microbial features recognized by plant immune receptors or disrupt signaling pathways that would otherwise trigger defense responses (Wei et al., 2018).

The Type III secretion system (T3SS) is a hallmark of *Ralstonia solanacearum* pathogenicity, enabling the direct injection of effector proteins into host cells. These effectors interfere with immune signaling, suppressing defense gene expression and promoting successful colonization. This molecular syringe delivers effector proteins into host cells, where they disrupt key signaling pathways involved in plant immune responses, such as salicylic acid (SA) and jasmonic acid (JA) signaling. Effector proteins not only suppress host defenses but also modulate cellular processes to favor bacterial colonization and spread. Variability in effector repertoires across strains presents challenges and opportunities for developing resistant crop varieties. For example, some effectors may inhibit the synthesis or signaling of salicylic acid, a key hormone involved in the plant defense against pathogens. Others may interfere with the jasmonic acid (JA) pathway, a key regulator of plant defense against necrotrophic pathogens and insect herbivores, as well as responses to environmental stresses (Shi et al., 2023; An and Zhang, 2024).

The strategic suppression of these pathways not only prevents the plant from mounting an effective defense response but also creates a more favorable environment for the bacterium to colonize and spread (Bentham et al., 2020). Understanding these evasion tactics provides critical insights into the pathogenicity of *Ralstonia solanacearum* and highlights potential targets for enhancing plant immunity or developing new antimicrobial strategies. Researchers continue to investigate the full spectrum of interactions between the pathogen’s effector proteins and host cellular machinery, aiming to identify novel approaches to protect plants from this devastating disease (Alonso-Diáz et al., 2021). The manipulation of host signaling pathways is another critical mechanism by which *Ralstonia solanacearum* undermines plant defenses. By altering hormonal pathways that regulate immune responses, *Ralstonia solanacearum* creates an internal environment that favors infection. This manipulation can suppress defense signaling and may increase nutrient availability within host tissues. A key target is the salicylic acid (SA) pathway, which is crucial for activating resistance against biotrophic pathogens (Tanaka et al., 2015).

*Ralstonia solanacearum* employs several effector proteins delivered via its Type III secretion system to inhibit salicylic acid (SA) production and signaling, thereby suppressing the host’s primary defense against bacterial invasion (Lu et al., 2018; Bentham et al., 2020). The pathogen also disrupts the jasmonic acid (JA) and ethylene signaling pathways, which are crucial for defense against necrotrophic pathogens and for regulating physiological responses. These manipulations create a hormonal imbalance, skewing host defenses away from effective pathogen resistance and enabling bacterial colonization (Tanaka et al., 2015; Shi et al., 2023).

The impacts of these hormonal disruptions vary significantly among economically important host plants such as tomato, potato, and eggplant. For example, in tomatoes, the suppression of SA signaling by *R. solanacearum* has been linked to delayed activation of systemic acquired resistance, resulting in increased susceptibility and yield losses. In potatoes, the pathogen’s ability to interfere with JA signaling is particularly detrimental, as this pathway is critical for wound healing and tuber development, further compounding the effects of infection. Eggplants rely on ethylene-mediated responses to defend against biotic stress. When these pathways are disrupted by the pathogen’s effectors, the plants often show stunted growth and reduced fruit quality (Tanaka et al., 2015; Shi et al., 2023). These hormonal manipulations significantly reduce crop yields and market quality, highlighting the need to understand how *R. solanacearum* modulates its virulence mechanisms in response to specific host plant species. Future research should clarify the molecular basis of these interactions to enable host-specific resistance and targeted management strategies.

Studying the specific interactions between *Ralstonia solanacearum* effectors and plant hormonal pathways may lead to effective strategies for developing disease-resistant crops. By genetically engineering plants to maintain stable hormonal signaling in the presence of these effectors, or by using biotechnological approaches to enhance the native defense responses, new strategies can be devised to protect crops from this devastating pathogen. Understanding these intricate interactions highlights the complexity of plant-pathogen dynamics and supports the development of targeted strategies for crop protection.

## 7 Systemic spread within the host

Once *Ralstonia solanacearum* colonizes the xylem vessels through initial attachment and biofilm formation, it initiates systemic spread throughout the host plant. This movement is crucial for understanding the bacterium’s full pathogenic potential (Hamilton et al., 2024). Utilizing the xylem’s natural function of transporting water and nutrients, the bacteria disseminate efficiently through the vascular network. As they multiply within the xylem, they are propelled by water flow, reaching stems, leaves, and fruits (Hamilton et al., 2024). Virulence factors such as enzymes and toxins facilitate the invasion by breaking down plant cell walls and weakening immune responses. This enables *Ralstonia solanacearum* to colonize additional tissues more effectively. The rapid spread often outpaces the plant’s defense, leading to widespread wilting and eventual death characteristic of bacterial wilt. Additionally, biofilms block xylem vessels, exacerbating transport disruption and symptoms (Schellenberger et al., 2019).

Understanding the dynamics of this systemic spread highlights the aggressiveness of *Ralstonia solanacearum* and underscores the challenges in controlling the disease once established. Research focused on blocking bacterial spread either by targeting movement-related proteins or by strengthening the plant’s ability to seal infected xylem vessels could lead to effective strategies for controlling bacterial wilt (de Pedro-Jové et al., 2021).

The systemic infection caused by *Ralstonia solanacearum* severely impacts plant health and agricultural productivity. As the bacterium spreads through the xylem, it causes extensive disruption to the vascular system, drastically reducing water and nutrient transport. This manifests as wilting, the most visible symptom, but beneath the surface, the effects are more destructive. Infected plants often show stunted growth and a significant decline in photosynthetic efficiency due to reduced water availability and physiological stress (Wang et al., 2023b). The plant’s productivity is directly impacted, resulting in diminished yields and poor-quality fruits and vegetables. For commercial crops, this means economic losses due to lower marketable output and increased management costs. If left unchecked, the infection can lead to plant death, resulting in total crop loss. Furthermore, systemic infection complicates crop rotation and soil management strategies due to the bacterium’s persistence in soil and plant debris, posing risks for subsequent plantings (Jones, 2021).

Addressing the consequences of systemic infection involves controlling the spread of the bacterium and improving plant resilience. Breeding for resistance, enhancing plant immune responses through biotechnological means, and developing robust management practices are essential to mitigate this devastating pathogen’s effects. Ongoing research into how *Ralstonia solanacearum* affects plant systems provides hope for more effective strategies to protect crops and ensure sustainable agricultural productivity.

## 8 Host response to infection

The response of host plants to infection by *Ralstonia solanacearum* involves a complex array of stress-related pathways, highlighting the plant’s attempt to defend itself and mitigate damage. A key component of this response is the activation of reactive oxygen species (ROS). Typically associated with the plant’s defense mechanisms, ROS can have dual roles. On one hand, they are involved in signaling processes that trigger defense responses, including the strengthening of cell walls to prevent further bacterial spread. On the other hand, excessive ROS production can lead to oxidative stress, damaging cellular structures and exacerbating the symptoms of disease (Hendrich et al., 2023).

In addition to ROS, Hormonal changes, particularly alterations in stress-related hormones like salicylic acid (SA), jasmonic acid (JA), and ethylene, play a crucial role in a plant’s response to infection, with SA associated with systemic acquired resistance against biotrophic pathogens, while JA and ethylene are linked to responses against necrotrophic pathogens and wound healing (Xiao et al., 2023). When infected with *Ralstonia solanacearum*, plants exhibit changes in these hormonal pathways, potentially disrupting defense mechanisms. Studies on tobacco and eggplant infected with *R. solanacearum* revealed significant alterations in JA content and genes involved in the JA signaling pathway, indicating induced defense responses. Additionally, the proteome analysis of tobacco cultivars showed significant changes in SA, JA, and ethylene-related proteins in response to *R. solanacearum* infection, suggesting a regulatory role in defense responses (Khan et al., 2023). These findings highlight the intricate interplay between hormonal signaling and pathogen-induced stress responses, influencing plant defense mechanisms and susceptibility to bacterial colonization. The infection by *Ralstonia solanacearum* triggers significant physical and biochemical changes in host plants, which are manifestations of the plant’s struggle to combat the pathogen and survive. Physically, the most apparent change is the wilting and yellowing of leaves, which result from the blockage of xylem vessels by bacterial biofilms, severely restricting water and nutrient flow. This blockage not only leads to dehydration but also impairs photosynthesis, contributing to reduced growth and vitality of the plant (Bhatt et al., 2024).

Biochemically, the plant undergoes several modifications in response to the bacterial invasion. There is an increased production of defense-related enzymes such as phenylalanine ammonia-lyase (PAL), which is involved in the synthesis of phenolic compounds that strengthen plant cell walls and potentially inhibit bacterial growth. Additionally, plants may increase the synthesis of pathogenesis-related proteins and antimicrobial compounds, which are part of the plant’s innate immune response. These compounds aim to localize and contain the infection, preventing its spread to healthier tissues (Kaur et al., 2022).

The accumulation of signaling molecules like salicylic acid, jasmonic acid, and ethylene are also noted, which, as previously mentioned, play crucial roles in mediating the plant’s stress and defense responses. The cross-talk among these signaling pathways indicates a highly coordinated, though often pathogen-manipulated, response that aims to optimize the plant’s survival chances under attack. Studies focusing on these biochemical pathways can provide insights into the resilience mechanisms of plants and could lead to the development of biotechnological or chemical interventions that enhance these natural defenses, offering new ways to manage bacterial wilt more effectively.

## 9 Genetic mechanisms of resistance to *Ralstonia solanacearum* in major crops, enhancing plant immunity and disease management

Several resistance strategies against *Ralstonia solanacearum* have been identified through breeding programs and genetic studies, focusing on key resistance genes and plant immune responses.

### 9.1 Arabidopsis and model systems

In *Arabidopsis thaliana*, the RRS1-R gene, working together with RPS4, confers resistance to *Ralstonia solanacearum* by detecting specific effector proteins and triggering immune responses that restrict bacterial spread (Deslandes et al., 2002; Guo et al., 2021). Mutations in RRS1-R increase susceptibility, highlighting its essential role in defense against bacterial wilt.

### 9.2 Solanaceous crops (tomato, eggplant, and potato)

Recent studies have shown that introducing the *Arabidopsis* EFR (elongation factor-Tu receptor) gene into tomato (*Solanum lycopersicum*) significantly enhances resistance to *R. solanacearum*. EFR recognizes bacterial elongation factor-Tu (EF-Tu), triggering immune responses in the host plant (Bentham et al., 2020).

In potato (*Solanum tuberosum*), QTL mapping has identified resistance loci such as Rsto1, which confers partial resistance to *Ralstonia solanacearum* (Farag et al., 2015). Additionally, the NLR gene Rpi-blb2, originally identified in wild *Solanum* species, has been found to reduce bacterial colonization when overexpressed in susceptible potato varieties (Wang J. et al., 2018; Schenstnyi et al., 2022).

In eggplant (*Solanum melongena*), wild relatives like *Solanum torvum* have shown natural resistance to *R. solanacearum*, and this resistance is being introduced into commercial cultivars through breeding efforts (Xiao et al., 2023).

### 9.3 Rice and monocots

Although rice is not a typical host of *R. solanacearum*, overexpression of the Xa21 gene originally known for conferring resistance to *Xanthomonas oryzae* has been shown to enhance resistance to *R. solanacearum* in transgenic rice lines (Dai et al., 2023).

### 9.4 Advances in CRISPR and genetic engineering for resistance

The CRISPR-Cas9 system used to modify susceptibility genes in plants, thereby enhancing resistance to *R. solanacearum*. For example, knocking out the DMR6 gene in tomato has been shown to improve resistance to bacterial wilt (Faizal et al., 2024). Researchers are also working to combine multiple resistance QTLs into a single cultivar to achieve broad-spectrum resistance (Shawky et al., 2024). Future research should focus on validating these resistance mechanisms in diverse agricultural settings and integrating these genes into breeding programs to develop durable, high-yielding resistant cultivars.

In various plant species, including Arabidopsis *thaliana*, EIN2 (ETHYLENE INSENSITIVE 2) is a central component of the ethylene signaling pathway, crucial for activating various defense mechanisms against pathogens. Enhanced ethylene signaling can increase resistance by promoting defensive responses and strengthening cell walls (Li et al., 2021). WRKY transcription factors, such as WRKY11 and ERF15, modulate the expression of downstream defense-related genes, enhancing resistance to *Ralstonia solanacearum*. The Kyoto Encyclopedia of Genes and Genomes (KEGG) analysis demonstrated that glutathione metabolism and phenylpropanoid pathways are primary resistance pathways to *R. solanacearum* infection. In the resistant cultivar, differentially expressed genes (DEGs) encoding CYP450, TCM, CCoAOMT, 4CL, PAL, CCR, CSE, and CADH, involved in the synthesis of plant antitoxins such as flavonoids, stilbenoids, and lignins, were enriched in the phenylpropanoid pathway and upregulated at 3- and 7-days post-inoculation (dpi) (Li et al., 2021; Yan et al., 2022).

According to Chen et al. (2022), genomic analysis of *R. solanacearum* strain FJ1003 revealed that the genome comprises a chromosome, a megaplasmid, and a small plasmid. Evolutionary analysis indicated that FJ1003 belongs to phylotype I and contains 76 Type III Effectors (T3Es). The study highlighted extensive horizontal gene transfer, particularly noting the RS-T3E-Hyp14 effector present in both prophages and genomic islands. Knockout mutants of RS-T3E-Hyp14 showed significant reductions in pathogenicity and impaired colonization ability in the host. These findings provide valuable insights for controlling bacterial wilt in tobacco by targeting specific genetic functions of the pathogen.

In maize (*Zea mays*), ZmWAK (Wall-Associated Kinase) genes are involved in cell wall integrity and defense responses, recognizing pathogen-induced damage and activating immune responses. Enhanced expression of WAK genes strengthens defenses against various pathogens, including bacteria (Yang et al., 2019, 2021). ZmPR1 (Pathogenesis-Related Protein 1) is upregulated during pathogen attack, helping to limit pathogen spread. Overexpression of PR proteins improves resistance to bacterial infections (Ma et al., 2022)

In wheat (*Triticum aestivum*), TaPIMP1 enhances resistance to *Ralstonia solanacearum* and improves tolerance to drought and salt stresses. TaPIMP1 encodes a MYB protein with DNA binding domains and transcription activation domains. The TaPIMP1 transcript level is significantly upregulated by inoculation with *Bipolaris sorokiniana* and drought treatment (Liu et al., 2011). TaNPR1 (Nonexpressor of Pathogenesis-Related Genes 1) is a key regulator of systemic acquired resistance, modulating PR genes expression to enhance resistance against various pathogens (Liu et al., 2019).

In rice (*Oryza sativa*), the Xa21 gene provides broad-spectrum resistance against *Xanthomonas oryzae pv. oryzae*, the causative agent of bacterial blight, by recognizing pathogen-associated molecular patterns (PAMPs) (Zhang and Zhou, 2013). The mechanisms of Xa21 can be explored and adapted for resistance to *Ralstonia solanacearum*. The OsNPR1 gene, similar to its wheat counterpart, regulates systemic acquired resistance and enhances PR genes expression, significantly increasing resistance to bacterial pathogens, including Xanthomonas oryzae pv. oryzae (Xoo), the causative agent of rice bacterial blight (Dai et al., 2023).

In sugarcane (*Saccharum* spp.), the ScAOC1 gene regulates responses to various stresses, enhancing tolerance to pathogens like *R. solanacearum*. Overexpression of ScOPR1 in sugarcane increases resistance by accumulating jasmonic acid (JA), salicylic acid (SA), and glutathione S-transferase (GST), and upregulating hypersensitive response genes. Transcriptome analysis revealed that differentially expressed genes (DEGs) in ScOPR1 overexpressing (OE) plants were significantly enriched in hormone transduction signaling and plant-pathogen interaction pathways (Sun et al., 2020). ScWRKY3 negatively regulates defense responses, contrasting with the roles of ScAOC1 and ScOPR1. ShNPR1, a clade II NPR1-like gene, enhances resistance by upregulating defense responses when overexpressed (Wang L. et al., 2018).

Recent studies have identified candidate genes such as RKL1, IRE3, RACK1B, and PEX3 using global collections, validating them as susceptibility factors. Enhancing plant resistance to *R. solanacearum* involves breeding, genetic engineering, and biotechnological approaches like CRISPR/Cas9 to edit genomes and introduce resistance genes. Marker-assisted selection (MAS) accelerates the development of resistant varieties by identifying and selecting key genes (Demirjian et al., 2022).

To improve resistance in crops like maize, wheat, rice, and sugarcane, traditional breeding combined with MAS and genetic engineering can introduce and enhance resistance genes. Transgenic plants expressing PR proteins, receptor kinases, and other defense-related genes also contribute to plants that lead to enhanced resistance, ensuring better crop health and productivity (Table 2). Summary of genes associated with resistance to *Ralstonia solanacearum* and other pathogens in various plant species. The table lists the gene name, the species in which the gene is found, the function of the gene, its relevance to plant defense mechanisms, and the corresponding references. Key genes include those involved in recognizing pathogen effectors, regulating immune responses, and synthesizing plant antitoxins. The table highlights genes such as RRS1-R, which triggers immune responses to limit bacterial spread, and Erecta, which alters vascular properties to increase resistance. It also includes genes like Xa21 in rice and TaPIMP1 in wheat, known for providing broad-spectrum resistance. Recent studies have identified additional candidate genes like RKL1, IRE3, RACK1B, and PEX3 as susceptibility factors, offering potential targets for developing resistant crop varieties.

**TABLE 2:** Genes associated with resistance to *Ralstonia solanacearum* and other pathogens.

Gene	Species	Function	Relevance	References
RRS1-R and RPS4	Arabidopsis *thaliana*	Recognizes specific effectors from *R. solanacearum* and triggers immune responses	Limits bacterial spread by activating defense mechanisms	Deslandes et al., 2002; Guo et al., 2021
Erecta	Arabidopsis *thaliana*	Encodes a receptor-like kinase involved in stomatal development	Increases resistance by altering vascular properties	Jordá et al., 2016; Chen L. et al., 2023
Bs4 and bs4c	Tomato (*Solanum lycopersicum*) and pepper	Encodes a leucine-rich repeat receptor-like protein	Restricts bacterial multiplication and spread	Wang J. et al., 2018; Schenstnyi et al., 2022
NRC1	Tomato (*Solanum lycopersicum*)	Helper NLR protein that mediates immune responses	Amplifies defense signals and coordinates responses	Bentham et al., 2020
EIN2	Various	Central component of the ethylene signaling pathway	Enhances resistance by promoting defensive responses	Li et al., 2021
WRKY11, ERF15	Various	WRKY transcription factors involved in regulating defense responses	Modulate expression of defense-related genes, upregulated in resistant cultivars	Yan et al., 2022
PR5	Various	Pathogenesis-related protein, part of innate immune system	Upregulated in response to pathogen attack, strengthens defenses	Anisimova et al., 2021; Li et al., 2021
CYP450, TCM, CCoAOMT, 4CL, PAL, CCR, CSE, CADH	Various	Involved in synthesis of plant antitoxins such as flavonoids, stilbenoids, and lignins	Enriched in phenylpropanoid pathway, upregulated in resistant cultivars	Li et al., 2021; Yan et al., 2022
ZmWAK	Maize (*Zea mays*)	Involved in cell wall integrity and defense responses	Strengthens defenses against various pathogens	Yang et al., 2019
ZmPR1	Maize (*Zea mays*)	Pathogenesis-Related Protein involved in systemic acquired resistance	Improves resistance to bacterial infections	Ma et al., 2022
TaPIMP1	Wheat (*Triticum aestivum*)	Provides resistance to *Ralstonia solanacearum*	Enhances resistance against fungi and bacteria	Liu et al., 2011
TaNPR1	Wheat (*Triticum aestivum*)	Key regulator of systemic acquired resistance	Boosts overall immune response	Liu et al., 2019
AX21 and Xa21	Rice (*Oryza sativa*)	Provides broad-spectrum resistance against Xanthomonas oryzae pv. oryzae	Mechanisms can be adapted for resistance to *R. solanacearum*	Zhang and Zhou, 2013
OsNPR1	Rice (*Oryza sativa*)	Regulates systemic acquired resistance	Increases resistance to various bacterial pathogens	Dai et al., 2023
ScAOC1, ScOPR1 and ScWRKY3	Sugarcane (*Saccharum* spp.)	Pathogenesis-Related Protein upregulated in response to pathogen attack	Improves resistance to bacterial infections	Wang J. et al., 2018; Sun et al., 2020; Zou et al., 2024
*NPR1-like a and ShNPR1*	Sugarcane (*Saccharum* spp.)	salicylic acid (SA) and abscisic acid (ABA)	Regulator of plant defense signals	Zang et al., 2022
RKL1, IRE3, RACK1B, PEX3	Various	Candidate genes identified as susceptibility factors	Potential targets for developing resistance	Demirjian et al., 2022
GmFLS2a, GmFLS2b	Soybean (*Glycine max*)	Encode receptors with exceptional flg22-binding domains, enhancing pathogen perception	Enhanced plant resistance by recognizing polymorphic flg22 from *R. solanacearum*	Chen Y. et al., 2023
FLS2	*Arabidopsis thaliana*	Leucine-rich repeat-containing receptor-like kinase that perceives bacterial flagellin	Triggers PAMP-triggered immunity (PTI) against bacterial pathogens	Chen Y. et al., 2023
FJ1003 and RS-T3E-Hyp14	Tobacco	reduction in pathogenicity and impaired colonization ability in tobacco	Controlling bacterial wilt	Chen et al., 2022

## 10 Future research directions

Despite significant advances in our understanding of *Ralstonia solanacearum* and its host interactions, critical gaps remain particularly in deciphering effector-host target interactions, understanding environmental influences on disease dynamics, and improving resistance durability across diverse crop species. Addressing these challenges is essential for advancing the field and mitigating the global impact of bacterial wilt. Among these, understanding the molecular basis of host resistance stands out as a top priority. Research focused on identifying specific genes and signaling pathways that confer resistance to economically important crops, such as tomatoes, potatoes, and eggplants, is essential for breeding resilient varieties. Leveraging tools like CRISPR-Cas9 to enhance these traits could provide durable solutions to bacterial wilt.

Equally important is the development of field-deployable diagnostic tools that enable early detection of *R. solanacearum*. Rapid, accurate, and cost-effective diagnostic methods could empower farmers, especially in developing countries, to implement timely management strategies and reduce crop losses. For example, lateral flow immunoassays and portable genetic detection kits could revolutionize disease monitoring in resource-limited regions. Another area demanding urgent attention is the role of environmental factors in influencing both pathogen virulence and host susceptibility. Climate variability, soil health, and water management practices significantly impact disease dynamics, and understanding these interactions can inform integrated management strategies tailored to specific agroecological conditions. *R. solanacearum* thrives at 25–30°C, and rising global temperatures may expand its range into cooler regions, increasing disease incidence. Higher temperatures accelerate the pathogen’s lifecycle, leading to more severe and widespread infections. High humidity and heavy rainfall support pathogen survival and movement in waterlogged soils, while drought conditions weaken plant defenses and increase susceptibility. However, climate-related stresses such as high temperatures and drought can disrupt key defense pathways, including salicylic acid (SA) and jasmonic acid (JA) signaling, thereby weakening the plant’s resistance to infection. Developing climate-resilient crop varieties is vital to address these challenges. Understanding how shifting environmental factors alter disease dynamics will enable the development of predictive models and adaptive strategies for sustainable management of bacterial wilt.

Addressing these research priorities is essential to tackling global food security challenges. In developing countries, where bacterial wilt heavily impacts smallholder farmers, minimizing crop losses is vital for protecting livelihoods and promoting economic resilience. Addressing these gaps through interdisciplinary approaches that integrate molecular biology, plant breeding, environmental science, and socioeconomic analysis will not only deepen our understanding of *Ralstonia solanacearum* but also contribute to resilient and sustainable agricultural systems.

Finally, the interactions between *Ralstonia solanacearum* and other microbial communities in the soil and within the plant itself are not well understood. The microbiome can significantly influence the outcome of the infection process, and leveraging these interactions might offer novel approaches to disease prevention and control. Addressing these gaps through interdisciplinary research approaches that combine microbiology, plant science, environmental science, and bioinformatics could significantly advance our ability to predict, prevent, and control infections by *Ralstonia solanacearum*, ultimately leading to more sustainable agricultural practices.

A promising direction for future research is the development of disease-resistant plant varieties by leveraging detailed insights into the interaction mechanisms between *Ralstonia solanacearum* and its hosts. A deeper understanding of how this bacterium utilizes its effector proteins and other virulence factors to suppress plant immune responses and promote infection could pave the way for innovative genetic engineering and breeding strategies. For example, identifying specific host receptors that interact with the pathogen’s effectors may facilitate the creation of plant varieties that lack these receptors, thereby preventing pathogen entry or disrupting its ability to manipulate host processes.

Additionally, enhancing the plant’s own defense mechanisms through genetic modifications that boost the production of defensive enzymes and compounds or strengthen cell walls could provide another effective strategy for resistance. For example, increasing the expression of genes involved in the synthesis of lignin or other phenolic compounds could help fortify the plant’s physical barriers against bacterial invasion. Moreover, the application of CRISPR-Cas technology offers promising prospects for directly editing the plant genome to enhance resistance to *Ralstonia solanacearum*. By knocking out genes that are exploited by the pathogen to establish infection or by introducing novel genes that confer resistance traits from other species, researchers can create plant varieties that are better equipped to withstand this devastating pathogen. The development of resistant varieties not only reduces the reliance on chemical controls but also contributes to more sustainable agricultural practices. Continued investment in research that integrates plant pathology, genetics, and molecular biology is essential to realize the full potential of these approaches. Such efforts will not only curb the impact of bacterial wilt but also enhance global food security in the face of increasing pathogen threats.

## 11 Conclusion

This review has comprehensively detailed the interactions between *Ralstonia solanacearum*, the causative agent of bacterial wilt, and its host plants. We have explored how this pathogen gains entry through natural openings and wounds, establishes itself by forming biofilms in the xylem, and utilizes a suite of virulence factors, including cell wall-degrading enzymes and effector proteins, to suppress and evade plant immune responses. The systemic spread of the bacterium throughout the plant exacerbates the severity of the disease, leading to significant impacts on plant health and agricultural productivity. The implications of understanding these interactions are profound for the management of bacterial wilt. By identifying the key mechanisms through which *Ralstonia solanacearum* interacts with plants, researchers and agriculturalists can develop more targeted strategies for disease control. These include breeding for resistant plant varieties, employing genetic engineering techniques to enhance plant defenses, and developing chemical treatments that disrupt critical pathogenic processes such as biofilm formation and effector protein function. Furthermore, the insights gained from studying these interactions offer potential for developing integrated disease management strategies that combine cultural practices, biological control, and the use of resistant varieties to manage bacterial wilt more effectively. As global agriculture continues to face challenges from plant diseases like bacterial wilt, the knowledge derived from such studies will be crucial in safeguarding crops and ensuring food security. In conclusion, continued research into the complex interactions between *Ralstonia solanacearum* and its host plants will not only deepen our understanding of plant pathology but also enhance our capability to develop innovative and sustainable solutions to combat this devastating pathogen.
